# Melatonin influence on miRNA expression in sperm, hypothalamus, pre-frontal cortex and cerebellum of Wistar rats

**DOI:** 10.1371/journal.pone.0312403

**Published:** 2025-01-27

**Authors:** Mísia Helena da Silva Ferro, Ingrid Morante, Fernanda Akane Nishino, Camila Estevam, Fernanda Gaspar do Amaral, José Cipolla-Neto, Taiza Stumpp

**Affiliations:** 1 Laboratory of Developmental Biology, Department of Morphology and Genetics–Paulista Medicine School, Federal University of Sao Paulo (UNIFESP), Sao Paulo, SP, Brazil; 2 Department of Physiology, Federal University of São Paulo (UNIFESP), São Paulo, SP, Brazil; 3 Department of Physiology and Biophysics, Neurobiology Lab, Institute of Biomedical Sciences, University of São Paulo (USP), São Paulo, SP, Brazil; Alexandria University, EGYPT

## Abstract

Melatonin is a pineal hormone synthesized exclusively at night, in several organisms. Its action on sperm is of particular interest, since they transfer genetic and epigenetic information to the offspring, including microRNAs, configuring a mechanism of paternal epigenetic inheritance. MicroRNAs are known to participate in a wide variety of mechanisms in basically all cells and tissues, including the brain and the sperm cells, which are known, respectively, to present 70% of all identified microRNAs and to transfer these molecules to the embryo. MicroRNAs from sperm have been associated with modulation of embryonic development and inheritance of psychiatric symptoms, including autism. Given that microRNAs and melatonin are ubiquitous molecules with important roles in the organism, the aim of this study was to investigate the expression of specific microRNAs in sperm, brain and cerebellum of pinealectomized rats. For this study, Wistar rats had their pineal gland removed at 60 *post-partum*. Part of these rats received exogenous melatonin until the day of the euthanasia. The control group did not receive any treatment or manipulation. The sperm, hypothalamus, prefrontal cortex and cerebellum were collected for analysis of microRNA expression by RT-qPCR. The results suggest that melatonin absence caused by pinealectomy increases the expression of the target microRNAs in the sperm. Although the data suggest an alteration (increase or decrease depending on the region and microRNA) of expression levels of some microRNAs in the brain and cerebellum of pinealectomized rats, the differences were not statistically significant. This seems to be a consequence of the intragroup variation. Melatonin administration restored the levels of the target microRNAs in the sperm. Additional studies are needed to understand the impact of the alterations of microRNA expression to the pinealectomized rats as well as to their descendants.

## Introduction

Melatonin, N-acetyl-5-methoxytryptamine, is a hormone produced in several organisms and synthesized exclusively at night. In mammals, melatonin is primarily produced by the pinealocytes, in the pineal gland, through the uptake of the amino acid tryptophan in the absence of light [1]. The pineal gland initiates melatonin synthesis under the command of a vast neural network that receives photoperiodic information from the environment. Due to the amphiphilic nature of melatonin, it is not stored and is destined directly for central and peripheral tissues. Melatonin concentration in plasma indicates the night period and allows physiological adaptations during the seasons [2]. Due to this function, it is referred as a chronobiotic substance [3].

Although melatonin is commonly referred to as the “sleep hormone”, it acts in a wide variety of physiological phenomena, working as an antioxidant and anti-inflammatory substance [4]. The neuroprotective role of melatonin, for example, has been reported in different psychiatric conditions, such as autism, ADHD, depression and anxiety [5–8], as well as in neuroplasticity [9]. The role of melatonin as an antidepressant has also been suggested, making it a possible adjuvant treatment to depression symptoms [10]. The molecular mechanisms involved in these melatonin actions in the brain include free radical scavenging as well as inhibition of pro-apoptotic factors and of pro-inflammatory substances such as interleukins. Melatonin action on neural stem cell proliferation and differentiation has also been documented [11, 12].

Some cellular actions of melatonin depend on specific cellular receptors. In humans, the MT1 (MTNR1A) and MT2 (MTNR1B) receptors are well known. Studies show that nuclear receptors for melatonin allow its action in the regulation of gene expression and genetic modulation [13]. Melatonin also influences epigenetic mechanisms in different cells and tissues by acting on microRNA (miRNA) expression, histone acetylation/phosphorylation and DNA methylation (Reviewed by [14]). Melatonin has been shown to act on the reduction of DNA-methyl transferases (DNMTs) expression, leading to a decrease of DNA methylation [15].

A recent study indicates that the pattern of melatonin is associated with the differential methylation of clock genes related to the circadian rhythm [16]. The interaction of melatonin with small non-coding RNAs (sncRNA) has also been reported in the regulation of its receptors [17], in its synthesis [18, 19], in spermatogenesis *in vitro* [20] and in various pathological conditions [21]. However, most data refer to exogenous melatonin. Little information is available about the action of endogenous melatonin on different cells and tissues that are responsive to exogenous melatonin.

MicroRNAs (miRNAs) are a type of sncRNA that control gene expression through pre and post-transcriptional mechanisms [22]. It has been shown that the brain expresses 70% of all identified miRNAs, illustrating their importance for this organ. Indeed, miRNAs control synapsis and neuron morphology, neurogenesis and neuron migration and metabolism. In addition, alterations in miRNA levels were detected in psychiatric and degenerative disorders. In the sperm cells, miRNA exact role has not been elucidated yet, although their importance to spermatogenesis and relationship with male infertility have been documented [23, 24]. It has also been shown that miRNA biogenesis occurs in the sperm and that these miRNAs are important to initial embryo development [25].

The influence of the administration of exogenous melatonin on the profile of miRNAs during spermatogenesis *in vitro* has been reported [20]. The action and influence of melatonin on spermatogenesis is of particular interest because the sperm transfer not only genetic but also epigenetic information to the embryo. Studies show that miRNAs are transferred from sperm to the embryo and can configure a mechanism of paternal epigenetic inheritance [26, 27]. In addition, some miRNAs whose expression is common to the brain and sperm, such as miR-34a and miR-132, have been related to the intergenerational transmission of neural and stress characteristics [28, 29]. Consequently, it is possible to hypothesize that sperm may also mediate the epigenetic inheritance of psychiatric disorders.

Considering that lifestyle has changed significantly in the last decades, leading to increased exposure to light, and that melatonin and miRNA act widely in the organism, we hypothesize that alterations in melatonin levels lead to alteration of miRNA levels in the brain and in the sperm. Thus, in view of the importance of miRNAs and melatonin for the central nervous system and the sperm, as well as the lack of information about the functions of endogenous melatonin, the objective of the present study was to evaluate the influence of melatonin absence on miRNA profile of sperm, hypothalamus, prefrontal cortex and cerebellum from pinealectomized rats.

## Materials and methods

### Animals and pinealectomy

For this study, 18 adults male Wistar rats (*Rattus norvegicus albinus*) were randomly distributed into three groups (n = 6): control, PINX and PINX-MEL. The PINX group consisted of pinealectomized rats without melatonin replacement. The PINX-MEL group was composed of pinealectomized rats that received melatonin replacement therapy. The animals in the control group were not pinealectomized and did not receive melatonin replacement. Surgery to remove the pineal gland was performed at 60 days of postnatal life (60dpn), the age at which the spermatogenesis is complete, and sperm have reached epididymis cauda. The sham group was not included in this study because it has been previously shown that the sham rats work like a control group [30–32]. All procedures were approved by the research ethics committee of the Federal University of São Paulo (CEUA N° 8074220415) and followed the recommendations of the International Standards for the Care and Use of Laboratory Animals.

The surgical technique of pineal removal described below is intended for experimentation with rats. The equipment and procedures carried out are part of the protocol intended for this species. The pinealectomy was performed at ZT 2 (abbreviation for Zeitgeber Time—time signal of the circadian system), that is, 2 hours after the lights were turned on in the animal facility. ZT 0 represents the beginning of the light phase, when the lights are turned on, while ZT 12 represents the beginning of the dark phase, when the lights go off. At the time of pineal removal, the rats were sedated and anesthetized using 10 mg/kg of xylazine and 40 mg/kg of ketamine. The animal was positioned in a stereotaxic apparatus in prone position and excess hair from the upper region of the head was removed. Using a scalpel blade, a longitudinal incision of approximately 2–3 cm was made in the skull and a circular trephine was used to expose the pineal gland, which was removed using forceps in a single movement. The blood was cleaned, and the removed cranial fragment was repositioned in the skull. Finally, the animals were placed back in the cages and remained under observation until their reflexes were recovered.

### Melatonin replacement therapy

PINX-MEL rats received melatonin (0.5mg/kg) that was added to water bottles during the dark phase of the light-dark cycle for 70 days from the same day of surgery to the euthanasia. This interval between pineal removal and euthanasia comprised at least one complete period of spermatogenesis, which lasts approximately 53 to 54 days in rats [16]. The dose of melatonin used in this study is higher than physiological concentrations (0.1mg/kg) to ensure that a basal concentration was consumed by each rat. Melatonin solution (Sigma-Aldrich, Cat. M5250) was prepared daily and administered at the beginning of the dark period (ZT 13), that is, 1 hour after the lights went off. Full access to the water bottle was allowed during the dark phase period. The rats were weighed weekly and the melatonin concentration for the PINX-MEL group was adjusted according to the average weight of the animals in each box and according to the daily water intake. The bottles with melatonin were removed one hour before the beginning of the light phase, at ZT 23 and replaced with bottles containing only water. As the half-life of melatonin is 20 minutes, the removal of the bottles with melatonin occurred at ZT 23 to ensure that the rats overcome the dark-light transition with ideal concentrations of melatonin. This process occurred every day until the day of euthanasia. Throughout the experiment, the animals were kept under controlled conditions of temperature (21 to 23°C), humidity and light (12h light/12h dark cycles).

### Euthanasia and sample collection

Euthanasia was carried out by decapitation at 130dpn and occurred at ZT 1. For sperm collection, the epididymis was isolated through abdominal incision. A small incision was made in the epididymis cauda to allow the contents to leak. The cauda was placed in a 1.5mL tube containing pre-heated Phosphate-Buffered Saline solution (PBS, pH 7.4) and incubated at 37°C for 10 minutes for sperm dispersion. The fragments of the epididymis cauda were removed and the solution containing the sperm was centrifuged. The pellet was resuspended in Somatic Cell Lysis Buffer Buffer (SCLB: 0.1% SDS and 0.5% Triton in ddH_2_O) on ice for 20 minutes, centrifuged for 15 minutes at 4°C and the supernatant was removed. This process was repeated, and the pellet was washed in 3mL of PBS to remove SCLB residues.

To collect the hypothalamus, prefrontal cortex and cerebellum, the encephalon was removed and placed in a Petri dish on ice; the tissues were directly allocated into 1.5mL tubes containing RNAlater Stabilization Solution® (ThermoFisher®) according to manufacturer specifications and stored at -70°C until the moment of RNA extraction.

### miRNA extraction and qPCR

The miRNA from sperm and brain regions was extracted using the mirVana® miRNA Isolation kit (Thermo Fischer Scientific, Cat. AM1560), according to the manufacturer instructions, except for the addition of a heating step (65°C for 10 minutes with low agitation) during incubation in Lysis Binding Buffer in the case of sperm samples. The synthesis of miRNA cDNA was performed using the TaqMan Advanced miRNA cDNA Synthesis® kit (ThermoFisher Scientific, Cat. A28007), according to the manufacturer instructions.

MiRNA expression was analyzed by RT-qPCR using the Roche LightCycler® 96 System platform. The target miRNAs analyzed were: miR-18a-5p, miR-34a-5p, miR-132-5p, miR-195-5p, miR-219a-5p, miR-182-5p and Let-7g-5p (Table 1). These target miRNAs were chosen based on data from the literature, which show that these miRNAs might be related to neurophysiology and/or sperm physiology. MiRNA Let-7a and Let-7g were used as reference for the data normalization from the hypothalamus, prefrontal cortex and cerebellum. MiRNA expression was analyzed using TaqMan assays (Thermofisher Scientific, Table 1). PCR conditions were used as follows: preincubation at 55° C for 120s (1 cycle), enzyme activation at 95°C for 20s (1 cycle), denaturation at 95°C for 3s, annealing/extension at 60°C for 30s (40 cycles). To perform the ΔCq calculations, the average of the Cq values of Let-7a and Let-7g was obtained. For data from sperm, the average of the Cq of all target miRNA combined was used. The expression level was obtained using the 2^-(ΔCq)^ method [33, 34]. All reactions were performed as duplicates and each reaction was repeated once. The data distribution was analyzed using the Shapiro-Wilk test. For those that showed normal distribution, the one-way ANOVA test was applied, followed by Tukey test when the differences were significant. For data that did not present a normal distribution, the Kruskal-Wallis multiple comparison test was applied, followed by Dunn test when significant differences occurred. The analyses were performed using the RStudio (version 4.3.1) program and differences were considered statistically significant when p ≤ 0.05.

**Table 1:** Target miRNA information.

Gene	Assay Name	Assay ID	miRNA Sequence
Let-7a	rno-let-7a-1-3p	rno481486_mir	CUAUACAAUCUACUGUCUUUCC
Let-7g-5p	rno-let-7g-5p	478580_mir	UGAGGUAGUAGUUUGUACAGUU
miR-18a-5p	rno-miR-18a-5p	rno480968_mir	UAAGGUGCAUCUAGUGCAGAUAG
miR-34a-5p	rno-miR-34a-5p	rno481304_mir	UGGCAGUGUCUUAGCUGGUUGU
miR-132-5p	rno-miR-132-5p	rno481320_mir	ACCGUGGCUUUCGAUUGUUACU
miR-182-5p	rno-miR-182-5p	477935_mir	UUUGGCAAUGGUAGAACUCACACU
miR-195-5p	rno-miR-195-5p	rno480882_mir	UAGCAGCACAGAAAUAUUGGC
miR-219a-5p	rno-miR-219a-5p	rno481348_mir	UGAUUGUCCAAACGCAAUUCU

### Biological pathways of the target miRNA

To obtain information about the possible biological meaning of the data obtained, the biological pathways of the miRNA were analyzed. First, the target genes of the miRNA were identified using the TargetScan (https://www.targetscan.org/vert_80/) and miRDB (https://mirdb.org/) databases. Then, the gene list resulting from TargetScan and miRDB was uploaded to Metascape database (https://metascape.org/) and heat maps indicating the enriched pathways for the target genes of the miRNAs of interest were generated.

## Results

### miRNA expression in sperm

A previous study carried out by our group (data not published) indicated that the Let-7g-5p miRNA is a good endogenous control for analyzing miRNA expression in brain and sperm samples. Furthermore, other studies [35–38] and data from the Spermbase platform (http://spermbase.org/) also indicate that this miRNA is a good reference option. However, in the present study it was observed that the absence of the pineal led to changes in the Cq of Let-7g in the sperm in relation to the control group, impeding this miRNA from being used as a reference for calculating ΔCq. Likewise, the Cq of all other target miRNAs in this study were also altered in the sperm, making their use as a reference miRNA unfeasible. Two other miRNAs, Let-7a and Let-7c, were also investigated as possible reference miRNAs for sperm analysis, but no consistent amplification was detected in the samples in this study. Thus, for relative expression analyses, the global average of Cq values obtained from all target miRNAs, including Let-7g, was used as the reference value to obtain ΔCq for each target miRNA [39].

**Fig 1:**
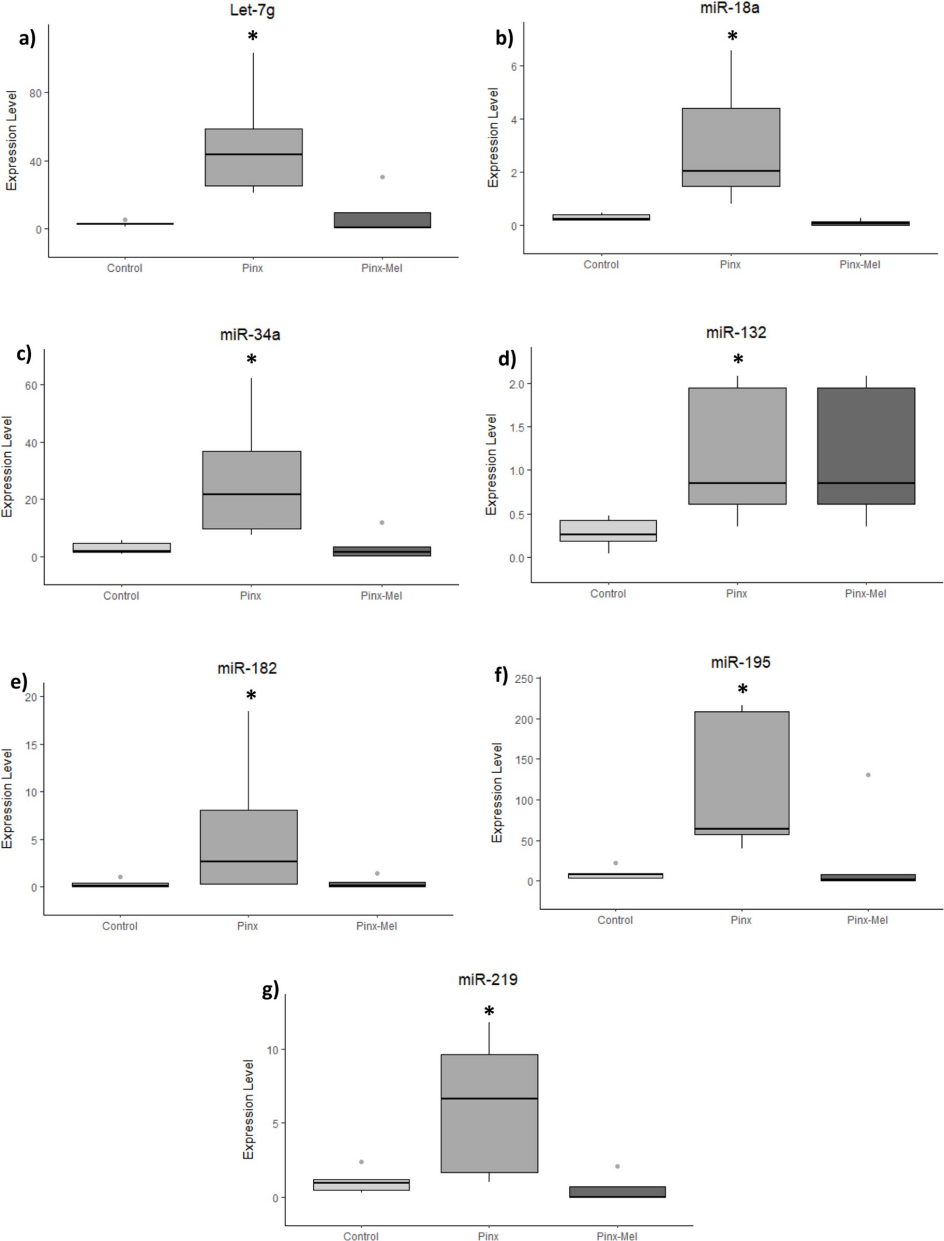
Expression level (2^-ΔCq^) of microRNA in the sperm of control, pinealectomized (PINX) and melatonin-treated (PINX-MEL) rats. In the sperm, all miRNA analyzed showed increased expression in the PINX group when compared with the control group. Melatonin administration led to a reduction in the expression of these miRNAs towards the values observed in control rats.

The expression level of all miRNAs (miR-18a, miR-34a-5p, miR-132-5p, miR-195-5p, miR-219a-5p, miR-182-5p and Let-7g-5p) was altered in the sperm of the PINX group (Fig 1). The miRNA that showed the most pronounced change was miR-182, followed by Let-7g and miR-195, while miR-132 showed the lowest degree of change. The administration of exogenous melatonin led to a reduction of the expression of all miRNAs analyzed in the sperm, leading the values to become close to the expression in the control group (Fig 1).

### miRNA expression in hypothalamus, prefrontal cortex and cerebellum

**Fig 2:**
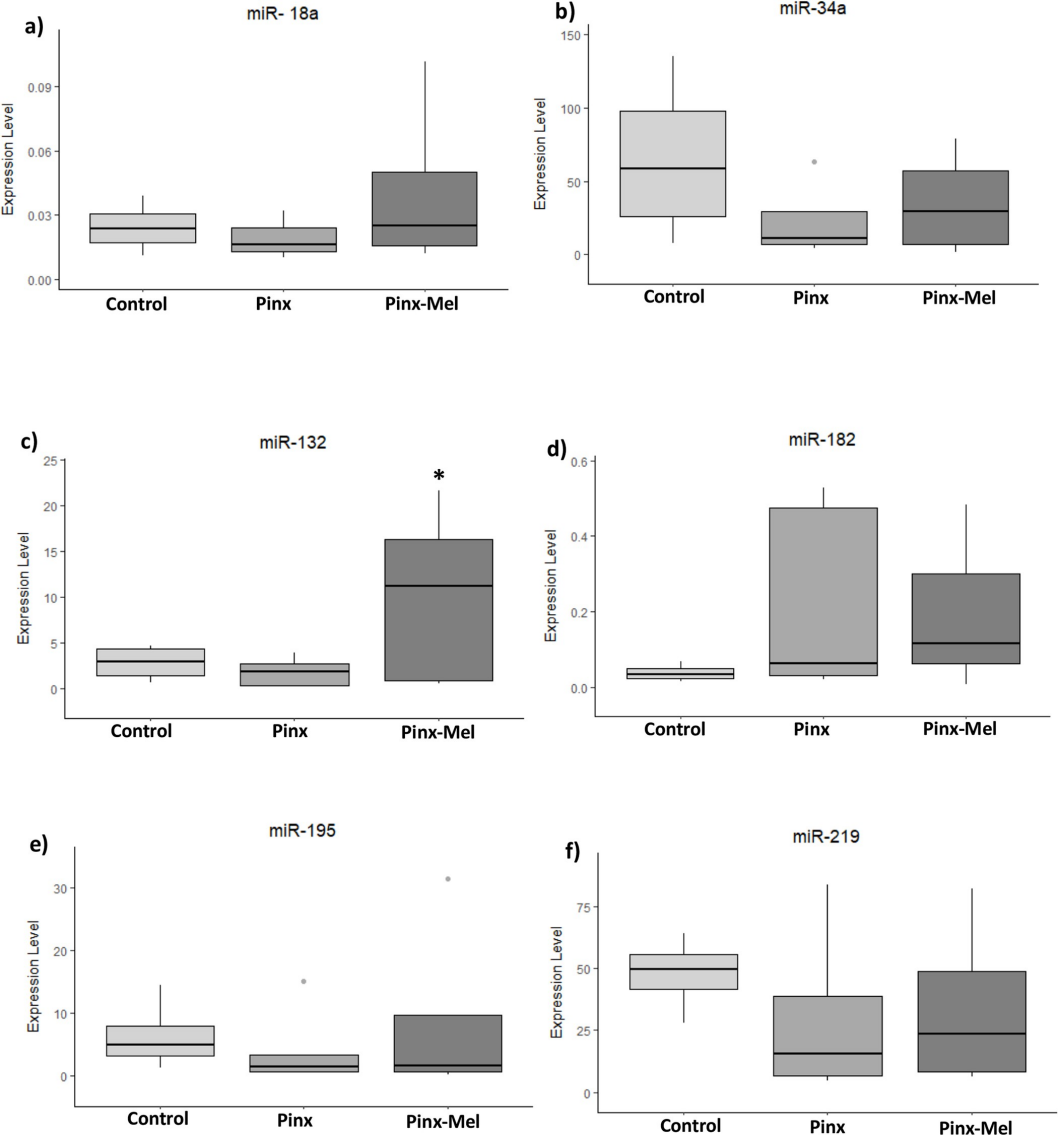
Expression level (2^-ΔCq^) of microRNA in the hypothalamus of control, pinealectomized (PINX) and melatonin-treated (PINX-MEL) rats. Although the differences were not significant, a tendency of increase in the expression of miR-34a, miR-132, miR-195 and miR-219 can be noticed in the hypothalamus of the PINX group. A significant increase of miR-132 expression was observed in the PINX-MEL group (Fig 2C, asterisk).

**Fig 3:**
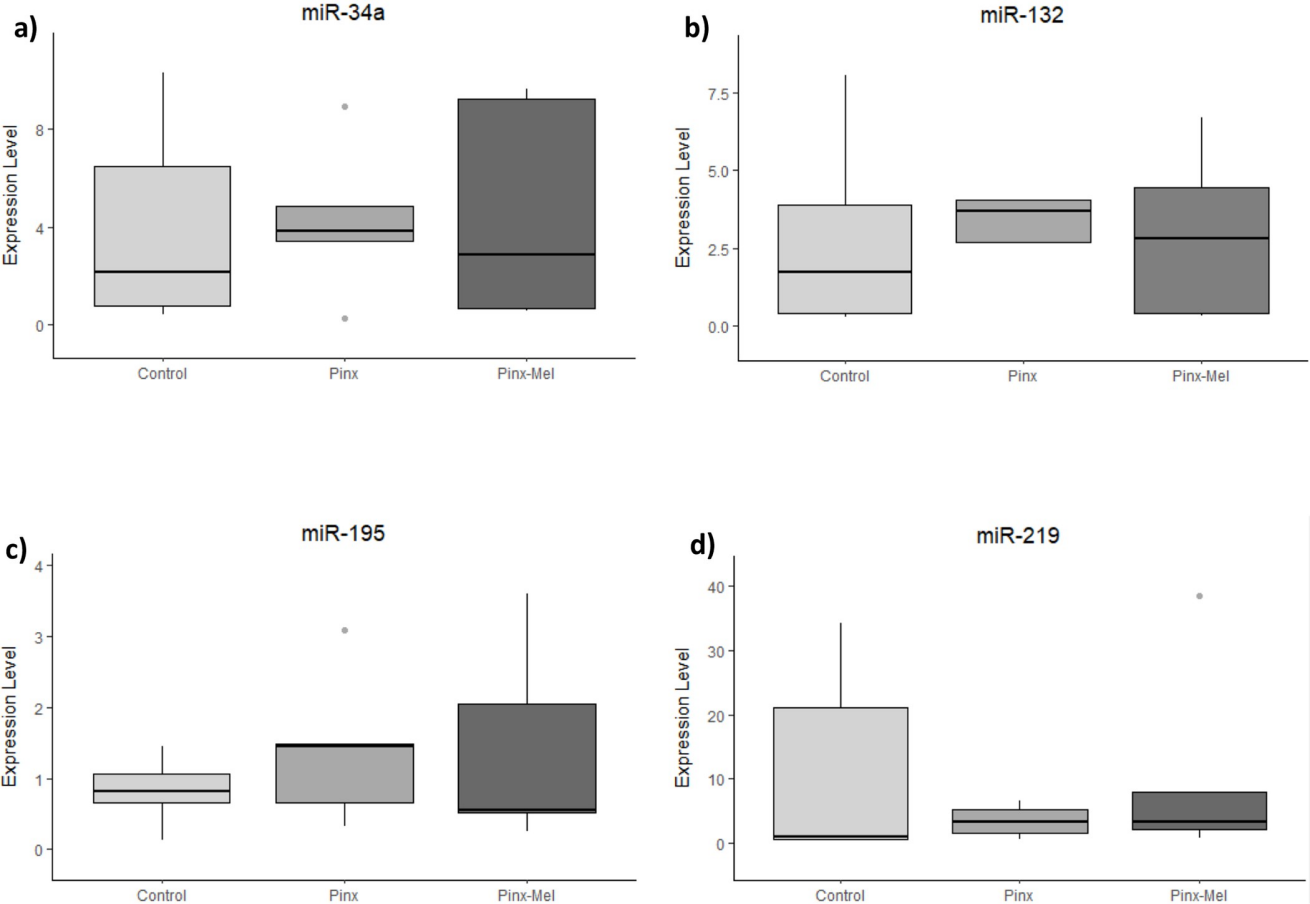
Expression level (2^-ΔCq^) of microRNA in the prefrontal cortex of control, pinealectomized (PINX) and melatonin-treated (PINX-MEL) rats. As observed for the hypothalamus, no significant difference was observed among the groups, although a tendency of increase in the expression of miR-34a, miR-132, miR-195 and miR-219 can be noticed in the PINX group.

**Fig 4:**
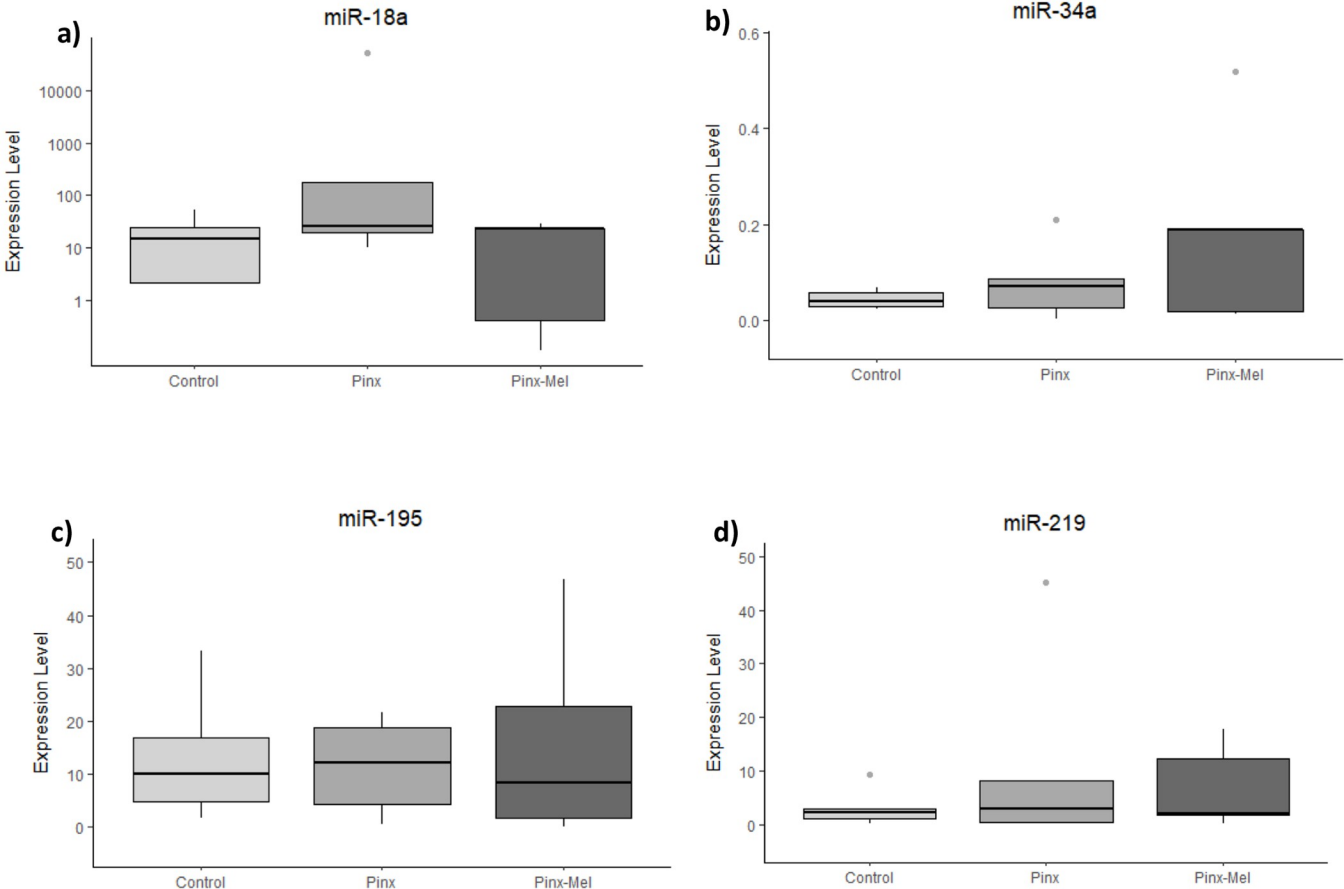
Expression level (2^-ΔCq^) of microRNA in the cerebellum of control, pinealectomized (PINX) and melatonin-treated (PINX-MEL) rats. As observed for the hypothalamus and prefrontal cortex, no significant difference was observed among the groups.

The remotion of the pineal gland caused no significant alteration in miRNA expression in the hypothalamus (Fig 2), prefrontal cortex (Fig 3) and cerebellum (Fig 4), although a decreasing tendency was observed for miR-34a, miR-132, miR-195 and miR-219 in the hypothalamus (Fig 2), whereas an increasing tendency was observed as well as for miR-34a and miR-132 in the prefrontal cortex of the PINX group (Fig 3). The administration of exogenous melatonin led to a pronounced and statistically significant increase of the expression of miR-132 in the hypothalamus of PINX-MEL group when compared to the PINX group (Fig 2). In the prefrontal cortex the expression of miR-132 showed a tendency of decrease after melatonin administration to pinealectomized rats (Fig 3). In the cerebellum no alteration of miRNA expression was detected after melatonin administration (Fig 4). MiR-18a and miR-182 were not detected in the prefrontal cortex and in the cerebellum.

### Enriched biological pathways

**Fig 5:**
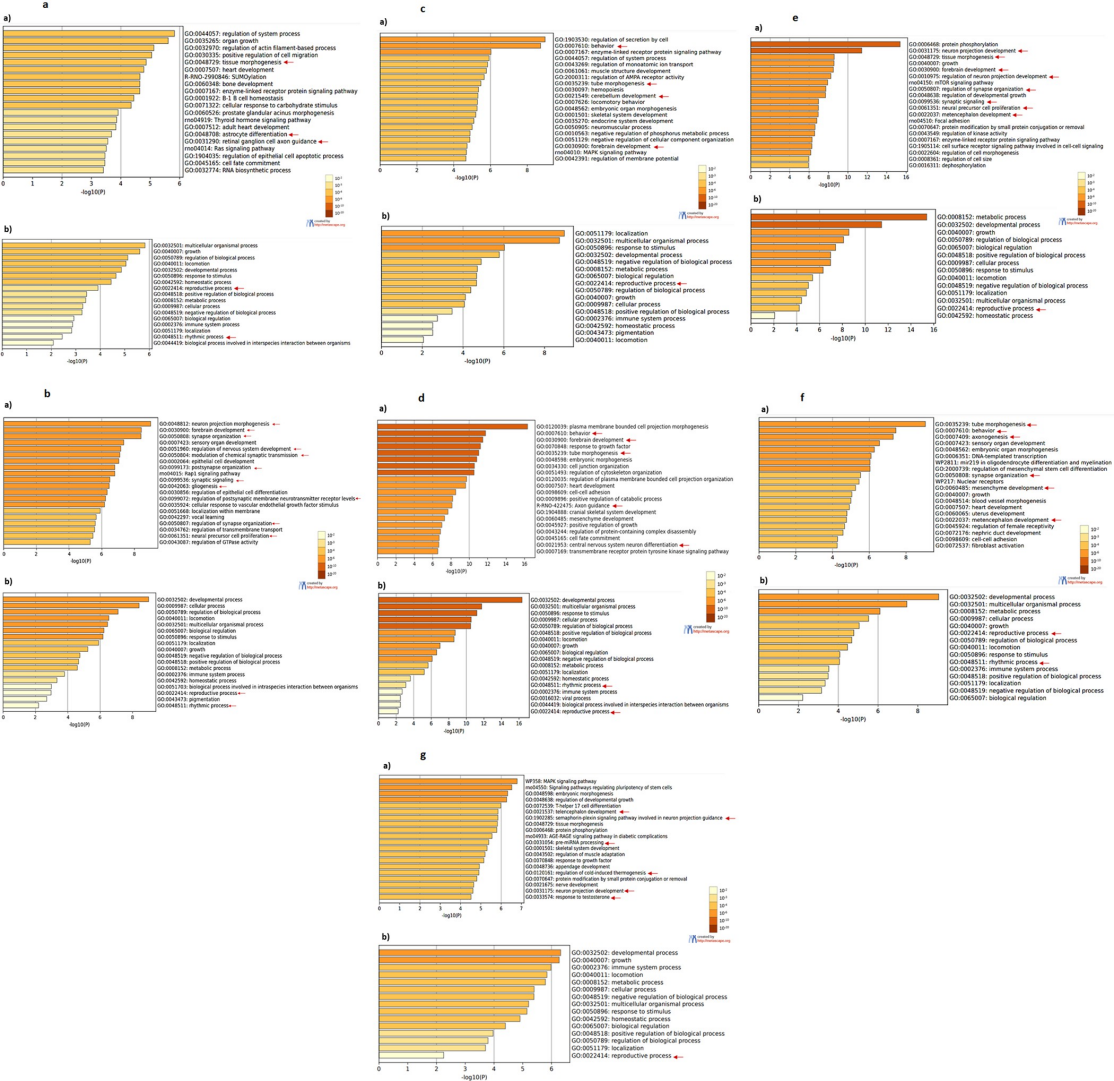
a: Heat map showing the enriched pathways (a) and ontology analysis (b) for miR-18a target genes. Although the enriched pathways do not show biological processes of interest (a), the gene ontology analysis (b) indicate the involvement of miR-18a in reproductive and rhythmic processes (arrows) among the 20 most enriched processes. b: Heat map showing the enriched pathways (a) and ontology analysis (b) for miR-34a target genes. The analysis returned processes related to neurodevelopment and neurophysiology (arrows) among the top 20 most enriched pathways (a). The gene ontology analysis (b) indicates the participation of genes targeted by miR-34a in rhythmic and reproductive processes (arrows). c: Heat map showing the enriched pathways (a) and ontology analysis (b) for miR-132 target genes. The top 20 enriched pathways (a) returned for miR-132 include neurodevelopment processes as well as behavior (arrows). The gene ontology analysis (b) show that the genes targeted by miR-132 are related to reproductive processes (arrow). d: Heat map showing the enriched pathways (a) and ontology analysis (b) for miR-182 target genes. It is indicated that the genes targeted by miR-182 participate on processes related to neurodevelopment and behavior (a), whereas the gene ontology indicate their involvement with rhythmic and reproductive processes (b). e: Heat map showing the enriched pathways (a) and ontology analysis (b) for miR-195 target genes. The analysis returned processes related to neurodevelopment (arrows) among the top 20 most enriched pathways (a). In the gene ontology analysis (b), the involvement of mIR-195 targeted genes in reproductive processes (arrow). f: Heat map showing the enriched pathways (a) and ontology analysis (b) for miR-219a target genes. The top 20 enriched pathways (a) returned for miR-219a include neurodevelopment and neurophysiology processes (arrows), whereas the gene ontology (b) indicate the involvement of genes targeted by miR-219a with rhythmic and reproductive processes (arrows). g: Heat map showing the enriched pathways (a) and ontology analysis (b) for Let-7g target genes. It is indicated that the genes targeted by Let-7g are involved in neurodevelopment (arrows), whereas the gene ontology indicate the participation of the Let-7g targeted genes in reproductive processes (arrow).

To obtain information about the biological meaning of changes in miRNA expression, bioinformatics analyzes were carried out using consolidated databases such as miRDB, TargetScan and Metascape. This investigation generated heat maps of enriched pathways for genes targeted by the miRNA studied here. The 20 most enriched pathways (top 20 clusters) generated by Metascape were considered (Fig 5A–5G). The total number of clusters generated for the genes targeted by each miRNA is presented as supplementary material (S1–S7 Figs).

Among the top 20 clusters, several biological pathways such as rhythmic processes, phenomena related to the central nervous system and its development, as well as behavior and reproductive processes (Fig 5A–5G) are indicated as a result of the Metascape analysis. In this analysis, miR-132 stands out, showing greater relevance for related processes (Fig 5C). Table 2 presents the miRNAs and the pathways in which they are potentially involved.

**Table 2:** Pathways with potential involvement of the target miRNAs.

Target miRNA	Potentially involved pathways
miR-18a	Retinal ganglion cell axon guidance and astrocyte differentiation.
miR-34a	Neuronal projection morphogenesis, forebrain development, regulation of nervous system development, gliogenesis, neural precursor cell proliferation, synaptic organization and regulation, modulation of chemical synaptic transmission, postsynapse organization, synaptic signaling, and regulation of neurotransmitter receptor levels of the postsynaptic membrane.
miR-132	Tubular morphogenesis, development of the cerebellum and forebrain, and involvement with target genes associated with behavior.
miR-182	Tubular morphogenesis, forebrain development, axon guidance and differentiation of neurons in the central nervous system.
miR-195	Development of neuronal projection and forebrain, proliferation of neural precursor cells, development of the hindbrain, regulation of synaptic organization and synaptic signaling.
miR-219a	Tube morphogenesis, axonogenesis, oligodendrocyte differentiation and myelination, hindbrain development, synapse organization, and target genes associated with behavior.
Let-7g	Telencephalon development, semaphorin-plexin signaling pathway involved in neuronal projection guidance, nerve development, neuronal projection development, response to testosterone, regulation of cold-induced thermogenesis and pre-miRNA processing.

## Discussion

MiRNA are subject of numerous studies that aim to elucidate epigenetic mechanisms involved in biological phenomena, including those related to the nervous system and gametes [40, 41] given the importance of these molecules for neurodevelopment and gametogenesis. Like miRNAs, melatonin has been identified as a molecule that performs important functions in the brain, providing protection against oxidative stress [42], neuronal apoptosis and neuroinflammation [43]. In this study, miRNAs that target genes related to neurogenesis, neurodevelopment, synaptogenesis, behavior modulation, circadian rhythm and other processes involved with the nervous system were analyzed [44, 45]. Data obtained from RT-qPCR suggest that melatonin absence caused by pineal removal led to changes in the expression of all analyzed miRNAs in sperm and of specific miRNAs in the hypothalamus, prefrontal cortex and cerebellum. Previous studies have shown that pinealectomized rats that did not receive melatonin replacement present desynchronization of the biological clock rhythm in Leydig cells due to rhythmic disorganization [46, 47], which agrees with the alteration in the expression of the miRNAs miR-34a, miR-132 and miR-195 in the brain, which are involved in the regulation of the circadian rhythm [48, 49].

The role of miRNAs in melatonin synthesis [19] as well as melatonin influence on miRNA expression has been previously reported [50–52]; however, the direct relationship of this hormone or its deficiency with the levels of miRNA in different tissues has been little explored so far, and the data that are available are still indirect and/or controversial [53]. Thus, the action of melatonin on miRNA expression has been inferred based on data about the role of these miRNAs in each tissue and cell, that is, from the understanding of the independent function of miRNAs and melatonin in these tissues and cells. The role of exogenous melatonin administration has also been explored due to increasing data about its functions and to the increasing use of this hormone as a dietary supplement. Studies show that miRNAs participate in different mechanisms by which exogenous melatonin acts on cellular stress [54], cognitive [55] and general inflammatory processes [56], in Alzheimer’s [52] and in memory [57]. Thus, the existence of a relationship between melatonin and miRNAs in neurophysiological processes seems clear. On the other hand, the meaning and the implications of the changes in miRNA expression observed here still need to be clarified.

RT-qPCR data showed that removal of the pineal gland led to a more marked alteration in the expression of miRNAs in sperm than in the brain and cerebellum. This data suggest that melatonin has remarkable influence on the physiology of these gametes. The effects of exogenous melatonin on general male reproductive parameters have been explored under different conditions and in different species. Its administration can improve spermatogenesis and fertility after exposure to toxic substances [58–64], but the molecular mechanisms by which melatonin acts on sperm are still unknown. *In vitro* studies suggest that melatonin acts on spermatogonia lineages by regulating the expression of miRNAs [20, 65]. However, no studies were found in the literature that investigated the influence of melatonin on sperm miRNA expression or during spermatogenesis *in vivo*; so, to the best of our knowledge, this is the first study to explore this relationship. In addition to the implications for their own physiology, changes in the expression of sperm miRNAs raise the question about their possible implications for embryonic development and about the possibility of transmitting these changes to descendants through epigenetic inheritance. Studies show that changes in the sperm miRNA profile can be transmitted to future generations [66–68]; so, it is reasonable to consider that the extensive change observed in the present study can potentially influence the next generation.

It is important to highlight that the administration of exogenous melatonin led to a relevant recovery in the expression of miRNAs in sperm, but not in all the nervous tissues analyzed. This data is consistent with those from the pinealectomized rats, reinforcing that sperm are very sensitive to melatonin, responding intensely to both the lack of it and its replacement. This is in line with the various studies that show that melatonin acts positively on different physiological aspects of sperm, leading, for example, to an improvement of their quality in *in vitro* fertilization protocols in different species, including humans [69–72].

Although the biological response to the changes in miRNA levels needs investigation, the high susceptibility of sperm to melatonin draws attention and suggests that this topic requires consideration, given the function of these cells to transmit genetic and epigenetic characteristics. To evaluate the possible biological meanings of the changes in the expression of miRNAs observed in this study, bioinformatics tools based on consolidated databases were used. The genes targeted by the miRNAs studied here were involved in biological processes related to the physiology and/or development of the central nervous system, such as synaptic organization, differentiation of neurons and glial cells, development of the brain and cerebellum and behavior. These results were expected, since the miRNAs analyzed in this study were chosen based on their relationship with neurodevelopment and neurophysiology. In addition to the relationship with neurodevelopment and neurophysiology, bioinformatics revealed that the genes targeted by miR-34a, miR-18a, miR-182 and miR-219, such as *Per1*, *Clock* and *Cry2*, are also involved in rhythmic processes, suggesting that these miRNAs play a role in these processes. Although the bioinformatic analysis has suggested the involvement of the miRNAs studied here in reproductive processes, the relevance with which these processes appear is low. This may be due to the lack of data about the action of miRNAs on sperm in these databases. Therefore, it is important to assess the implications of the changes observed here for these gametes and for the embryos generated by them.

The variety of processes, tissues and cells highlighted by bioinformatics analysis reinforces the ubiquitous characteristic of melatonin and miRNAs in the body, which explains the broad alteration in the expression of miRNAs observed in this study, especially in sperm, but also in the brain and cerebellum. This ubiquitous characteristic makes data interpretation quite complex and challenging. In this context, it was surprising that melatonin absence caused more pronounced changes in the expression of miRNAs in sperm than in the brain and cerebellum, since the functions of melatonin in neurophysiology are much better documented than in sperm. On the other hand, the central nervous system is considered the richest in diversity of miRNAs in comparison to other systems of the organism [73], what, combined with the multiplicity of genes targeted by these miRNAs, might suggest that the functions of these miRNAs coincide with other miRNA, generating mechanisms of compensation which prevent an imbalance in the synthesis of these molecules.

Considering that epigenetic inheritance is a relatively new topic and that very little is known about this subject, further studies are of great importance, especially given the high and indiscriminate consumption of melatonin throughout the world. Added to this is the fact that melatonin is being used to improve some undesirable symptoms that are more common in psychiatric disorders, such as difficulty sleeping, for example [74]. Much progress has been made in knowledge about psychiatric disorders and their heredity has been the subject of many studies. Thus, the relationship between melatonin and the brain in different conditions and the epigenetic characteristics of sperm emerges as an important aspect to be considered in the approach to psychiatric disorders and their inheritance.

In conclusion, data of the present study suggest that melatonin deficiency influence the expression of miRNAs related to neurodevelopment and neurophysiology in sperm and, to a lesser extent, in the brain and cerebellum. Additional studies are needed to better explore these results and to clarify the biological meanings of these changes for pinealectomized animals and whether they can have an impact on their offspring.

## Supporting information

S1 Fig Expanded heat maps showing the enriched pathways. The pathways for the genes targeted by miR-18a are presented.
(TIF)

S2 Fig Expanded heat maps showing the enriched pathways. The pathways for the genes targeted by miR-34a are presented.
(TIF)

S3 Fig Expanded heat maps showing the enriched pathways. The pathways for the genes targeted by miR-132 are presented.
(TIF)

S4 Fig Expanded heat maps showing the enriched pathways. The pathways for the genes targeted by miR-182 are presented.
(TIF)

S5 Fig Expanded heat maps showing the enriched pathways. The pathways for the genes targeted by miR-195 are presented.
(TIF)

S6 Fig Expanded heat maps showing the enriched pathways. The pathways for the genes targeted by miR-219a are presented.
(TIF)

S7 Fig Expanded heat maps showing the enriched pathways. The pathways for the genes targeted by Let-7g are presented.
(TIF)

S1 File Raw data. Details of qPCR results and statistical analysis.
(XLSX)
