# Supplementary figures and images for: Melatonin influence on miRNA expression in sperm, hypothalamus, pre-frontal cortex and cerebellum of Wistar rats
Source: PLoS One. 2025 Jan 27;20(1):e0312403. doi: 10.1371/journal.pone.0312403 (PMC11771911; doi:10.1371/journal.pone.0312403)

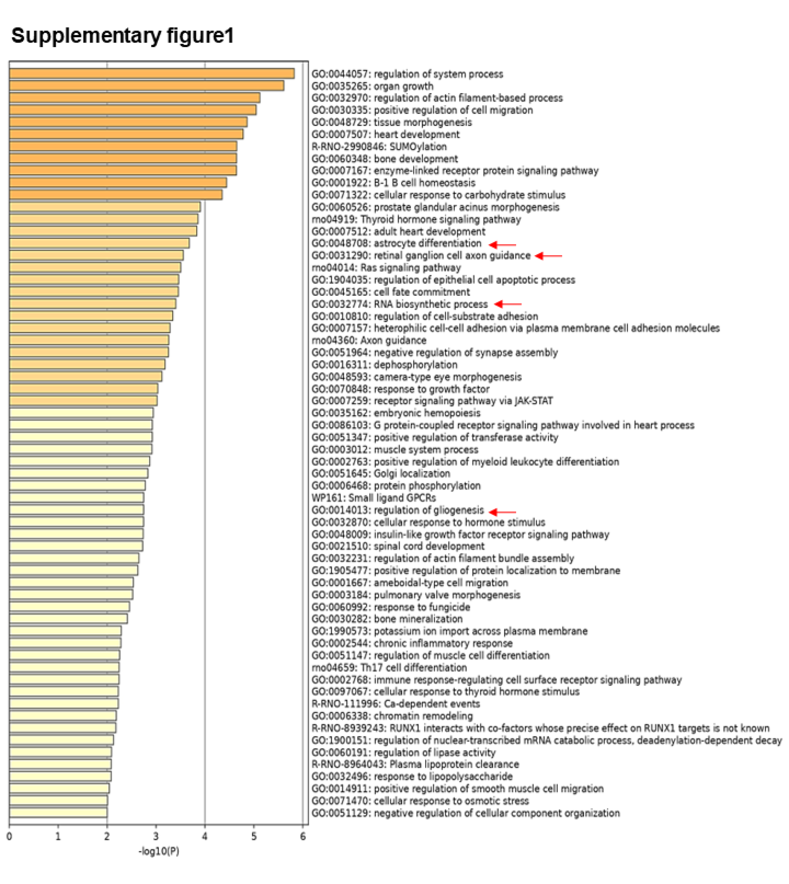

Supplement: S1 Fig — The pathways for the genes targeted by miR-18a are presented. (TIF) [file pone.0312403.s001.tif]

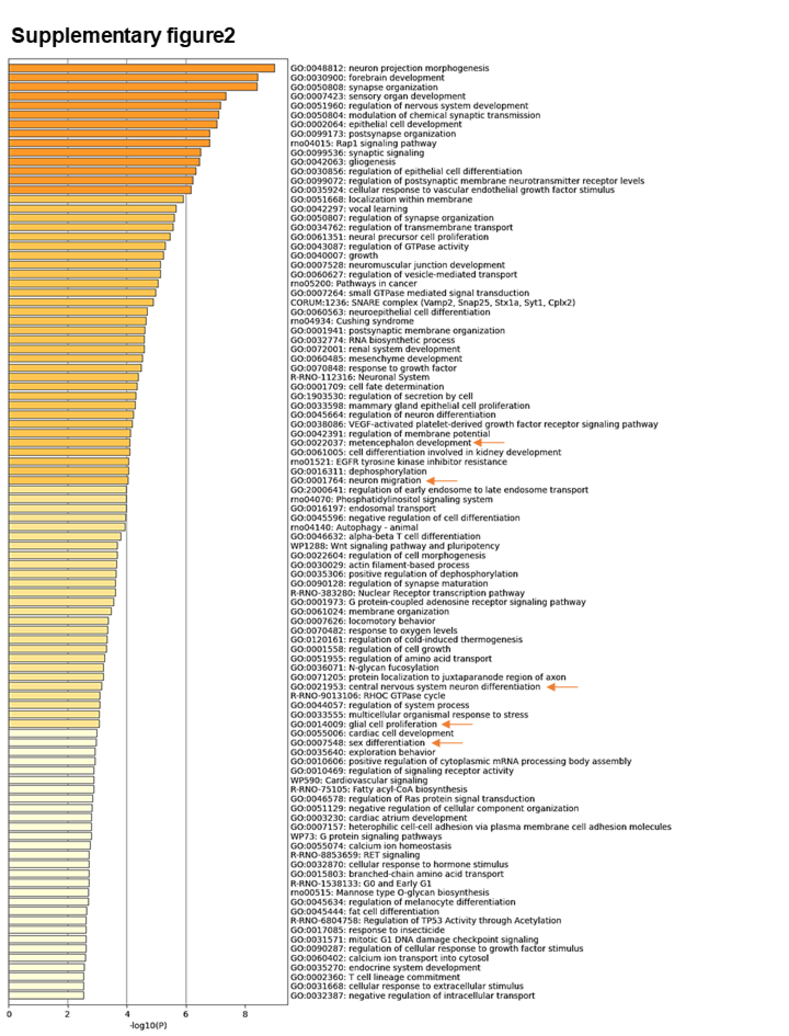

Supplement: S2 Fig — The pathways for the genes targeted by miR-34a are presented. (TIF) [file pone.0312403.s002.tif]

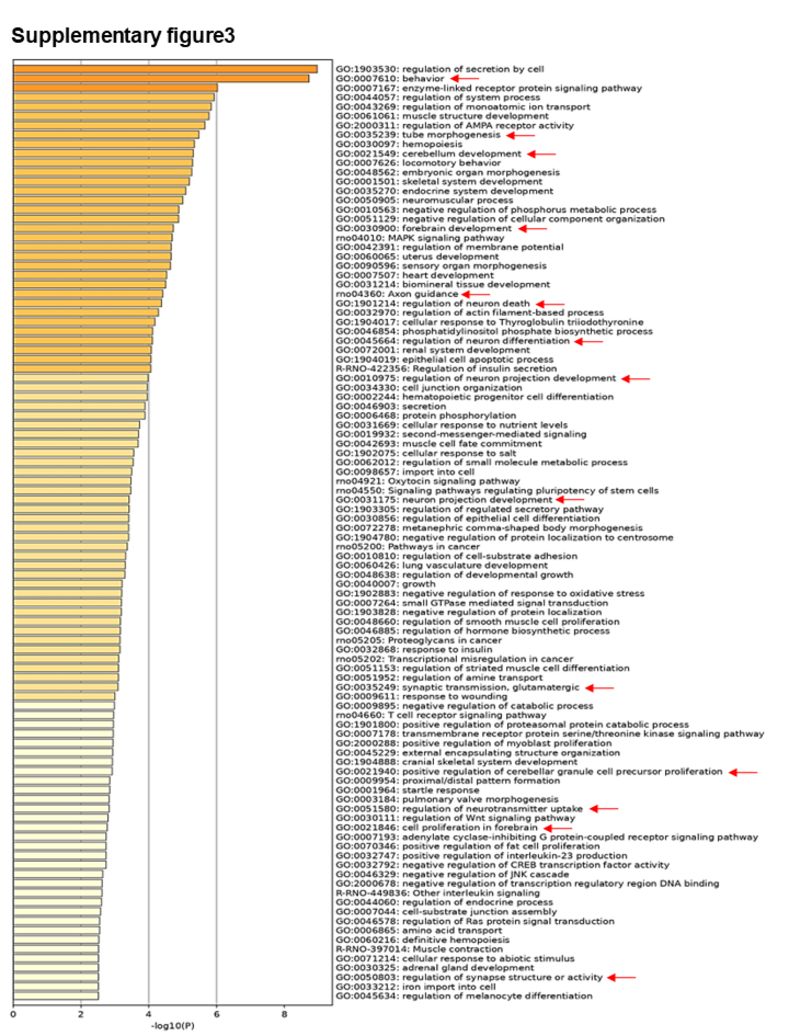

Supplement: S3 Fig — The pathways for the genes targeted by miR-132 are presented. (TIF) [file pone.0312403.s003.tif]

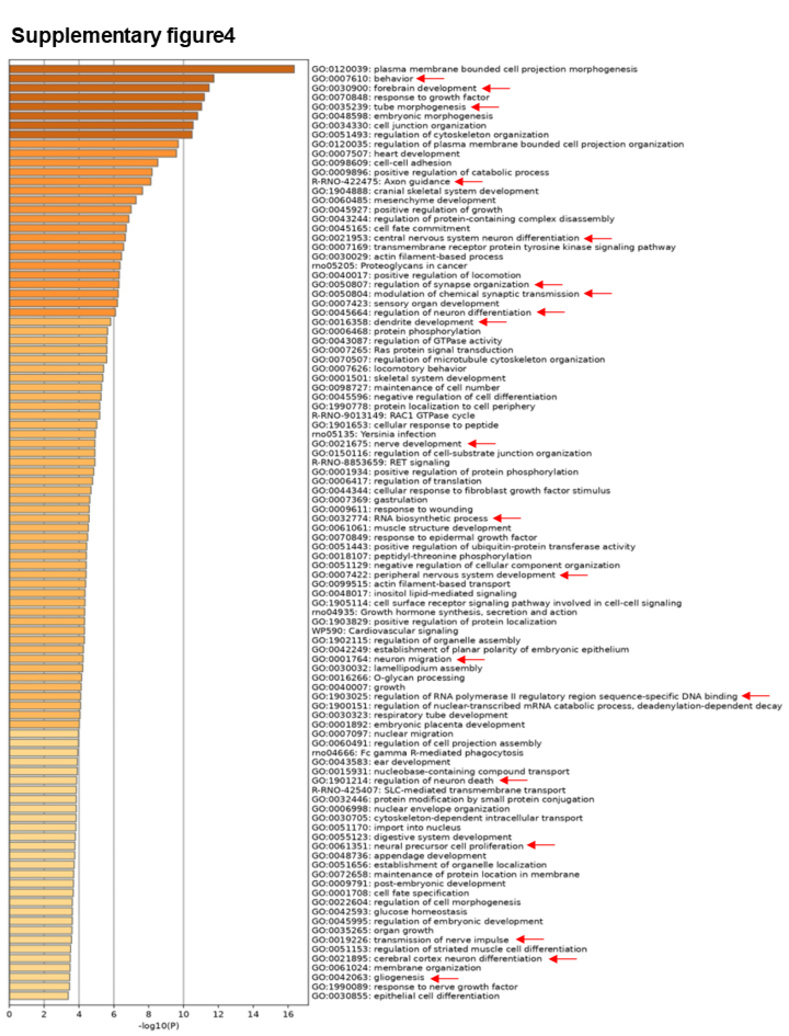

Supplement: S4 Fig — The pathways for the genes targeted by miR-182 are presented. (TIF) [file pone.0312403.s004.tif]

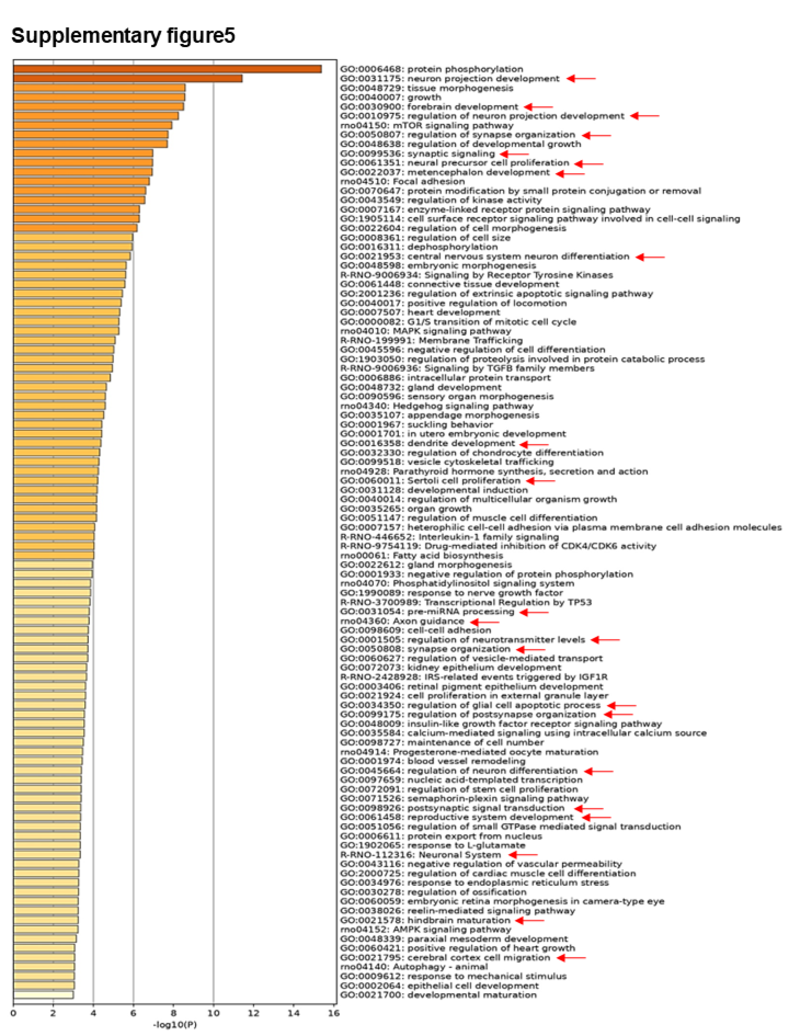

Supplement: S5 Fig — The pathways for the genes targeted by miR-195 are presented. (TIF) [file pone.0312403.s005.tif]

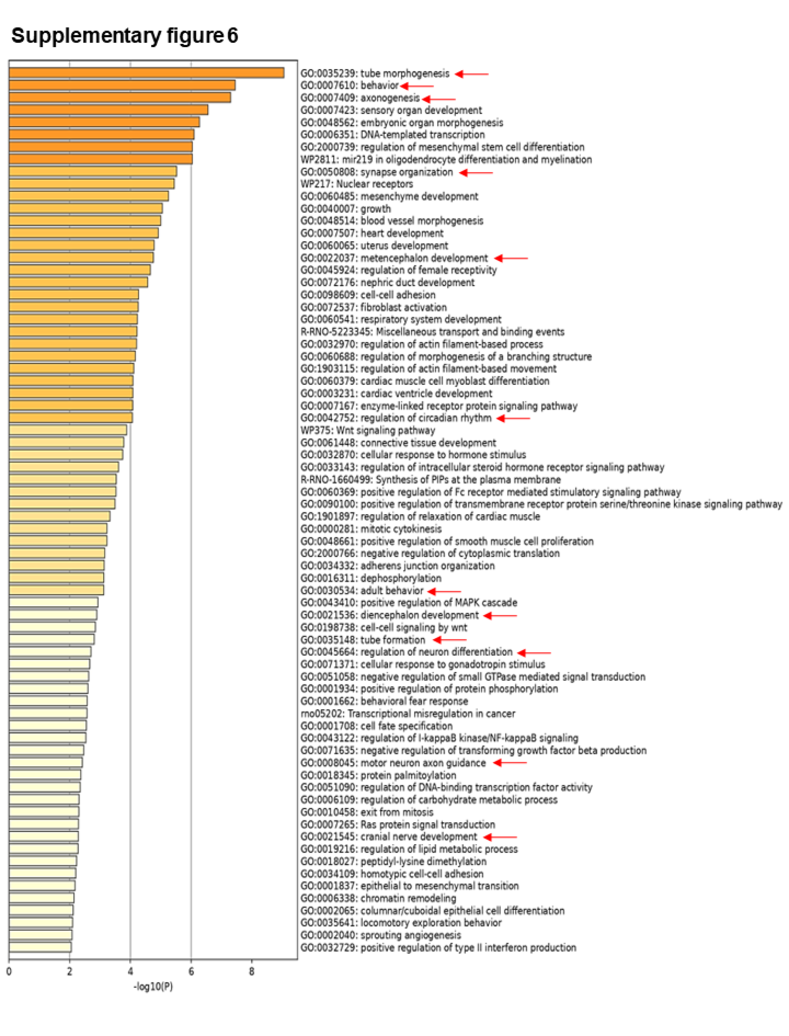

Supplement: S6 Fig — The pathways for the genes targeted by miR-219a are presented. (TIF) [file pone.0312403.s006.tif]

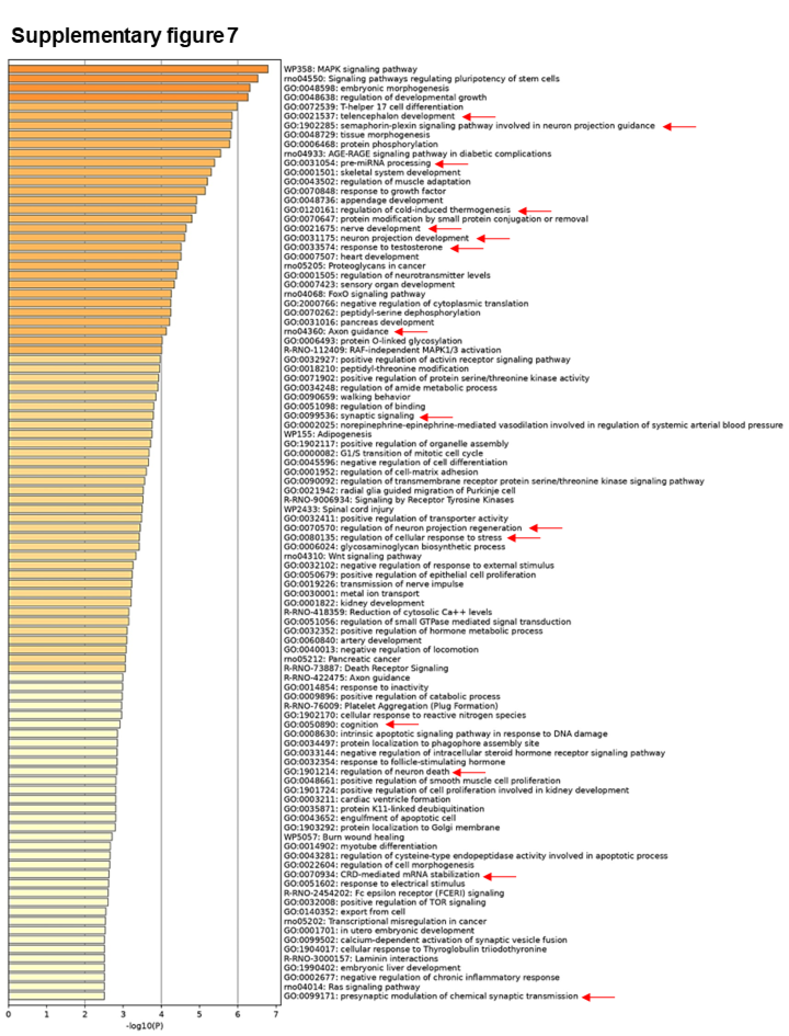

Supplement: S7 Fig — The pathways for the genes targeted by Let-7g are presented. (TIF) [file pone.0312403.s007.tif]
